# Sex differences in body composition and association with cardiometabolic risk

**DOI:** 10.1186/s13293-018-0189-3

**Published:** 2018-06-27

**Authors:** Melanie Schorr, Laura E. Dichtel, Anu V. Gerweck, Ruben D. Valera, Martin Torriani, Karen K. Miller, Miriam A. Bredella

**Affiliations:** 1 Neuroendocrine Unit, Massachusetts General Hospital and Harvard Medical School, Bulfinch 457B, 55 Fruit Street, Boston, MA 02114 USA; 2 Department of Radiology, Massachusetts General Hospital and Harvard Medical School, Yawkey 6E, 55 Fruit Street, Boston, MA 02114 USA

**Keywords:** Obesity, Body composition, Sex characteristics, Metabolic syndrome, Magnetic resonance spectroscopy, Computed tomography

## Abstract

**Background:**

Body composition differs between men and women, with women having proportionally more fat mass and men more muscle mass. Although men and women are both susceptible to obesity, health consequences differ between the sexes. The purpose of our study was to assess sex differences in body composition using anatomic and functional imaging techniques, and its relationship to cardiometabolic risk markers in subjects with overweight/obesity.

**Methods:**

After written informed consent, we prospectively recruited 208 subjects with overweight/obesity who were otherwise healthy (94 men, 114 women, age 37 ± 10 years, BMI 35 ± 6 kg/m^2^). Subjects underwent dual-energy X-ray absorptiometry (DXA) and computed tomography (CT) for fat and muscle mass, proton MR spectroscopy (1H-MRS) for intrahepatic (IHL) and intramyocellular lipids (IMCL), an oral glucose tolerance test, serum insulin, lipids, and inflammatory markers. Men and women were compared by Wilcoxon signed rank test. Linear correlation and multivariate analyses between body composition and cardiometabolic risk markers were performed.

**Results:**

Women and men were of similar mean age and BMI (*p* ≥ 0.2). Women had higher %fat mass, extremity fat, and lower lean mass compared to men (*p* ≤ 0.0005). However, men had higher visceral adipose tissue (VAT) and IMCL and higher age-and BMI-adjusted IHL (*p* < 0.05). At similar age and BMI, men had a more detrimental cardiometabolic risk profile compared to women (*p* < 0.01). However, VAT in women, and IMCL in men, were more strongly associated with cardiometabolic risk markers, while more lower extremity fat was associated with a more favorable cardiometabolic profile in women compared to men (*p* ≤ 0.03).

**Conclusions:**

Although the male pattern of fat distribution is associated with a more detrimental cardiometabolic risk profile compared to women of similar age and BMI, VAT is more strongly associated with cardiometabolic risk markers in women, while IMCL are more detrimental in men. Lower extremity fat is relatively protective, in women more than in men. This suggests that detailed anatomic and functional imaging, rather than BMI, provides a more complete understanding of metabolic risk associated with sex differences in fat distribution.

## Background

Body composition differs between men and women, with women having proportionally more fat mass and men more muscle mass [1, 2]. Although men and women are both susceptible to obesity, health consequences differ between the sexes [3]. Men have higher cardiovascular mortality, but women have a greater increase in cardiovascular mortality as BMI or waist circumference increases [4, 5]. This may be in part related to sex-specific differences in fat accumulation as the distribution of fat has a greater impact on cardiometabolic risk than excess total fat mass. For example, ectopic fat depots, such as visceral adipose tissue (VAT), intramyocellular lipids (IMCL), and intrahepatic lipids (IHL), are major risk factors for insulin resistance, dyslipidemia, and the metabolic syndrome, while lower extremity fat may protect against cardiometabolic disease [6–8]. Dual-energy X-ray absorptiometry (DXA) can assess total body and appendicular fat and lean mass [9], while computed tomography (CT) and magnetic resonance imaging (MRI) are considered the gold standard for the quantification of different abdominal fat compartments, such as subcutaneous adipose tissue (SAT) and VAT [10, 11]. Proton magnetic resonance spectroscopy (1H-MRS) is able to determine the amount of IHL [12, 13] and IMCL [14, 15] non-invasively.

A study from the Framingham Heart cohort has demonstrated positive associations between abdominal adipose tissue compartments and measures of cardiometabolic risk, which were stronger in women compared to men, but the study did not examine associations with ectopic fat depots, such as IHL and IMCL, lower extremity fat or muscle mass [16]. Moreover, a study in subjects at risk for type 2 diabetes mellitus (T2DM) has revealed higher VAT, lower SAT, and higher IMCL in men compared to women; however, the study did not examine the relationship of body composition to markers of cardiometabolic risk [17].

Therefore, the purpose of our study was to assess sex differences in body composition and its relationship to cardiometabolic risk markers in men and women with overweight/obesity. We hypothesized that there are sex differences in body composition and ectopic fat depots and that these are associated with a sex-specific cardiometabolic risk profile.

## Methods

This prospective study was IRB-approved and was HIPAA-compliant. Data were acquired after written informed consent was obtained from all subjects prior to the study.

### Subjects

Our study was performed at a clinical research center. The study group was comprised of 208 subjects (94 men, 114 women) with overweight or obesity who were recruited over 8 years through advertisements for participation in two NIH-funded studies. Inclusion criteria for this analysis were all subjects aged 18 to 65 years. Exclusion criteria were use of anti-hypertensive or cholesterol medications, diabetes mellitus, liver disease or other chronic illnesses, smoking, estrogen or glucocorticoid use, and contraindications to MRI.

Participants underwent DXA, CT, and 1H-MRS for assessment of body composition and ectopic fat depots. An oral glucose tolerance test was performed in all subjects and fasting and 2-h glucose was assessed. Insulin was measured in 63 men and 73 women. The homeostasis model assessment of insulin resistance (HOMA-IR), a marker of insulin resistance, was assessed. Serum lipids [triglycerides, total, high density lipoprotein (HDL), and low-density lipoprotein (LDL) cholesterol] were measured in all subjects. Apolipoprotein B (ApoB) was measured in 59 men and 77 women, and ApoB/LDL, a marker of atherogenicity, was calculated. Inflammatory markers [high-sensitivity C-reactive protein (hsCRP) and fibrinogen] were measured in 59 men and 77 women. Metabolic syndrome was defined as by the National Cholesterol Education Program criteria (NCEP Adult Treatment Panel III) [18].

Main outcome measures (IHL and IMCL) have been previously reported in a subset of study subjects (62 men and 79 women) [12, 15, 19–23]; however, none of the clinical characteristics, measures of cardiometabolic risk, and body composition have been reported in the entire cohort and no sex differences in body composition have been assessed.

### Dual-energy x-ray absorptiometry (DXA)

Subjects underwent DXA (Discovery A; Hologic Inc.) for assessment of total fat mass, percent body fat (%fat), lower extremity fat mass, and appendicular lean mass. The relative amount of lower extremity fat was calculated as the ratio of lower extremity fat mass over total fat mass. The relative amount of appendicular lean mass was calculated as the ratio of appendicular lean mass over total body weight. Coefficients of variation (CV) of DXA in our laboratory are 1.7% for fat and 2.4% for lean soft tissue mass.

### Computed tomography (CT)

Subjects underwent single slice CT (LightSpeed Pro, GE Healthcare) of the abdomen through the mid-portion of the L4 level and the left mid-thigh. Measurements performed at the L4 level have been shown to correlate with abdominal adipose tissue volumes in men and women and with cardiometabolic risk [24, 25]. Scan parameters were standardized: 144 table height, 80 kV (abdomen), 120 kV (thigh), 70 mA (abdomen), 170 mA (thigh), gantry rotation time 2 s, 1 cm slice thickness, and 48 field of view.

Abdominal and thigh adipose tissues were identified using a threshold set for − 50 to − 250 Hounsfield units (HU) [26], and abdominal and thigh SAT, VAT, and thigh muscle cross-sectional areas (CSA) (cm^2^) were separated by manual delineation. VAT/SAT was calculated to assess the relative amount of VAT. Analyses were performed using Osirix software version 3.2.1 (www.osirix-viewer.com/index.html). CV of CT in our laboratory are 2.5% for fat and 1.1% for muscle CSA.

### Proton MR spectroscopy (1H-MRS)

Subjects underwent 1H-MRS of the liver to determine IHL and of the soleus muscle to determine IMCL after an overnight fast using a 3.0-T MRI system (Siemens Trio, Siemens Medical Systems). All subjects were asked to avoid moderate or vigorous exercise or high-fat diet 72 h prior to scanning. CV for measurements at our institution are 8% for IHL and 6% for IMCL quantification.

### 1H-MR spectroscopy of liver

For 1H-MRS of the liver, a voxel measuring 20 × 20 × 20 mm (8 mL) was placed within the right hepatic lobe, avoiding vessels or artifacts. For each voxel placement, automated optimization of gradient shimming was performed. Single breath-hold single-voxel 1H-MRS data were acquired using a point-resolved spatially localized spectroscopy (PRESS) pulse sequence without water suppression with the following parameters: TR of 1500 ms, TE of 30 ms, 8 acquisitions, 1024 data points, and receiver bandwidth of 2000 Hz.

### 1H-MR spectroscopy of soleus muscle

For 1H-MRS of soleus muscle, the right calf was placed in a transmit/receive quadrature extremity coil (USA Instruments, Aurora, Ohio). A voxel measuring 15 × 15 × 15 mm (3.4 mL) was placed in the soleus muscle, avoiding interstitial fat or vessels. Single-voxel 1H-MRS data was acquired using a PRESS pulse sequence with a TR of 3000 ms, TE of 30 ms, 64 acquisitions, 1024 data points, and receiver bandwidth of 1000 Hz. Frequency selective water signal suppression was used for metabolite acquisition, and unsuppressed water spectra of the same voxel were obtained for each scan with the same parameters as the metabolite acquisition except for the use of eight acquisitions. For each voxel placement, automated optimization of gradient shimming, water suppression, and transmit-receive gain were performed, followed by manual adjustment of gradient shimming targeting water linewidths of < 20 Hz.

### 1H-MR spectroscopy data analysis

Fitting of all 1H-MRS data was performed using LCModel (version 6.3-0K). Fitting algorithms specific for liver lipid estimates (0.9, 1.3, and 2.0 ppm) were scaled to unsuppressed water peak (4.7 ppm) and expressed as lipid-to-water ratio. For soleus muscle, IMCL (1.3 ppm) and EMCL (1.5 ppm) methylene estimates were automatically scaled to unsuppressed water peak (4.7 ppm) and expressed as lipid-to-water ratio.

### Statistical analysis

JMP Statistical Database Software (version 12; SAS Institute) was used for statistical analyses. Men and women were compared using the Wilcoxon signed rank test. The Bonferroni method was used to control for multiple comparisons. Multivariate standard least squares regression modeling was performed to control for age and BMI on log-transformed data. Linear correlation analyses between body composition and measures of cardiometabolic risk were performed and nonparametric Spearman’s rank correlation coefficients are reported. Data are shown as median and interquartile range. Statistical significance was defined as a two-tailed *p* < 0.05, and *p* ≤ 0.1 was used to denote a trend.

## Results

Clinical characteristics and sex differences in body composition as assessed by CT, DXA, and 1H-MRS are shown in Tables 1 and 2. Women and men were of similar age and BMI. As expected, men were on average heavier and taller compared to women resulting in similar BMI.

**Table 1:** Clinical characteristics and body composition (median, IQR)

Variable	Men (*n* = 94)	Women (*n* = 114)	*p*
Clinical characteristics			
Age (years)	34.5, 28.8–42.0	38.0, 29.8–43.3	0.2
Height (cm)	177, 173–183	163, 158–168	< 0.0001
Weight (kg)	105, 96–124	91, 78–101	< 0.0001
Body mass index (kg/m^2^)	34, 31–39	34, 29–38	0.4
DXA parameters			
Total fat mass (kg)	34, 29–48	38, 31–47	0.4^#^
Total fat mass (%)	33.4, 28.5–37.5	42.2, 38.3–45.9	< 0.0001*^#^
Lower extremity fat (kg)	12, 10–16	14, 11–18	0.001*^#^
Lower extremity fat/total fat	0.38, 0.35–0.42	0.33, 0.30–0.36	< 0.0001*^#^
Appendicular lean mass (kg)	33, 29–37	23, 20–25	< 0.0001*^#^
Appendicular lean mass/weight	0.30, 0.28–0.33	0.25, 0.23–0.27	< 0.0001*^#^
CT parameters			
Abdominal SAT (cm^2^)	386, 292–560	455, 325–567	0.07*^#^
Visceral adipose tissue (VAT) (cm^2^)	149, 122–208	106, 69–139	< 0.0001*^#^
VAT/SAT	0.36, 0.25–0.54	0.23, 0.17–0.33	< 0.0001*^#^
Thigh SAT (cm^2^)	128, 94–148	171, 136–233	< 0.0001*^#^
Thigh muscle (cm^2^)	198, 177–219	141, 124–153	< 0.0001*^#^
^1^H-MRS parameters			
Intrahepatic lipids (lipid/water)	0.06, 0.03–0.17	0.05, 0.02–0.13	0.1*^#^
Soleus intramyocellular lipids (lipid/water)	0.04, 0.02–0.05	0.03, 0.02–0.04	0.0005*^#^

*Abbreviations*: *DXA* dual energy x-ray absorptiometry, *CT* computed tomography, *SAT* subcutaneous adipose tissue, *1H-MRS* proton magnetic resonance spectroscopy

*Significant after controlling for age

^#^Significant after controlling for BMI

**Table 2:** Cardiometabolic risk parameters (median, IQR)

Variable	Men (*n* = 94)	Women (*n* = 114)	*p*
Glucose parameters			
Fasting glucose (mg/dL)	85, 80–90	86, 81–92	0.4
OGTT 2-h glucose (mg/dL)	114, 95–133	119, 98–134	0.6
Fasting insulin (uU/mL)	14, 9–18	8, 5–10	< 0.0001^†^*^#^
HOMA-IR	2.6, 1.7–3.7	1.5, 1.0–2.2	< 0.0001^†^*^#^
Lipid parameters			
Total cholesterol (mg/dL)	172, 151–194	177, 156–202	0.2
HDL cholesterol (mg/dL)	38, 33–43	51, 43–59	< 0.0001^†^*^#^
LDL cholesterol (mg/dL)	107, 93–125	106, 87–128	0.7
Triglyceride level (mg/dL)	111, 82–160	89, 69–127	0.001^†^*^#^
Apolipoprotein B (ApoB) (mg/dL)	95, 84–110	85, 69–106	0.01*^#^
ApoB/LDL	0.88, 0.83–0.98	0.79, 0.70–0.87	< 0.0001^†^*^#^
Inflammatory parameters			
hsCRP (ng/L)	2.4, 1.4–6.0	2.9, 1.3–5.5	0.8
Fibrinogen (mg/dL)	395, 344–452	475, 419–523	< 0.0001^†^*^#^
Metabolic syndrome (*n*, %)	34 (37%)	19 (17%)	0.002^†^

*Abbreviations*: *HOMA-IR* homeostatic model assessment of insulin resistance, *OGTT* oral glucose tolerance test, *HDL* high-density lipoprotein, *LDL* low-density lipoprotein, *hsCRP* high-sensitivity C-reactive protein

^†^Significant after controlling for multiple comparisons

*Significant after controlling for age

^#^Significant after controlling for BMI

### Sex differences in body composition as assessed by dual-energy x-ray absorptiometry  (DXA)

There was no significant difference in mean total fat mass by DXA between men and women. However, women had higher mean %fat mass and higher mean lower extremity fat and higher lower extremity fat/total fat mass compared to men, while men had higher mean appendicular lean mass and appendicular lean mass/weight. These differences were independent of age and BMI (Table 1).

### Sex differences in body composition as assessed by computed tomography (CT)

**Fig. 1:**
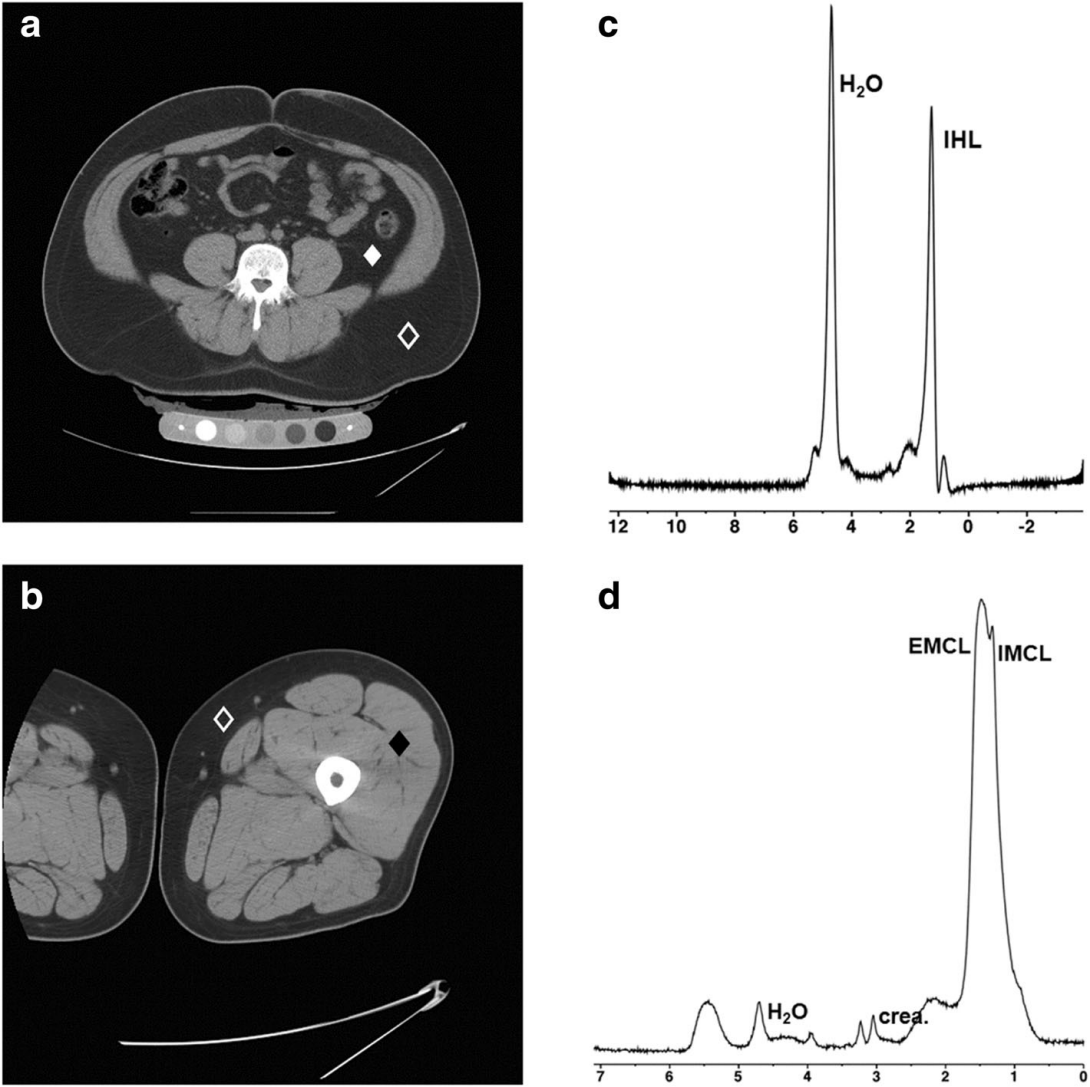
CT and 1H-MRS for body composition in a 35-year-old man with obesity (BMI 37 kg/m^2^). Fasting LDL cholesterol 118 mg/dL, HDL cholesterol 39 mg/dL, triglycerides 74 mg/dL, glucose 84 mg/dL, insulin 19 μU/mL, HOMA-IR 3.3, 2-h glucose from oral glucose tolerance test 144 mg/dL. **a** CT of the abdomen at the level of L4 for quantification of visceral adipose tissue (194 cm^2^) (white diamond) and subcutaneous adipose tissue (open diamond) (458 cm^2^). **b** CT of the mid-thigh for quantification of subcutaneous adipose tissue (open diamond) (123 cm^2^) and muscle (black diamond) (207 cm^2^). **c** 1H-MR spectrum of the right hepatic lobe for intrahepatic lipid quantification showing lipid (1.3 ppm) and unsuppressed water (4.7 ppm) resonances. Lipid to water ratio was 0.8. **d** 1H-MR spectrum of soleus muscle for intramyocellular lipid quantification showing intramyocellular lipid methylene protons (-CH2) at 1.3 ppm (IMCL), extramyocellular lipid methylene protons (-CH2) at 1.5 ppm (EMCL), residual water peak at 4.7 ppm, and creatine (-CH2) resonance at 3.0 ppm. IMCL/unsuppressed water ratio was 0.06

**Fig. 2:**
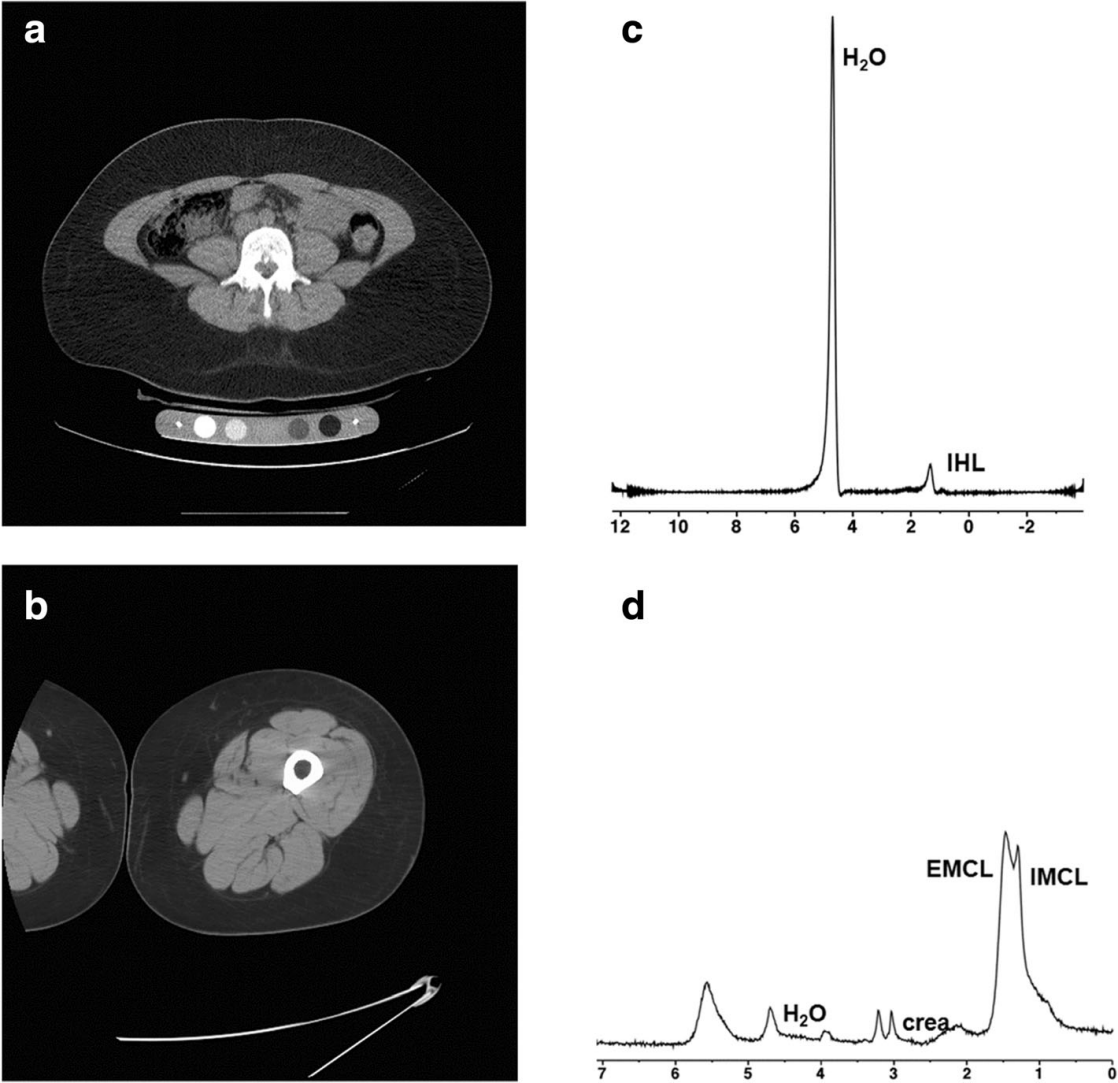
CT and 1H-MRS for body composition in a 35-year-old woman with obesity (BMI 38 kg/m^2^). Despite similar age and BMI, the woman had lower visceral adipose tissue and thigh muscle cross sectional area (CSA), lower intrahepatic and intramyocellular lipids and higher thigh subcutaneous adipose tissue compared to the man in Fig. 1, and this was associated with a more favorable cardiometabolic risk profile compared to the man. Fasting LDL cholesterol 104 mg/dL, HDL cholesterol 59 mg/dL, triglycerides 50 mg/dL, glucose 72 mg/dL, insulin 3 μU/mL, HOMA-IR 0.48, 2-h glucose from oral glucose tolerance test 84 mg/dL. **a** CT of the abdomen at the level of L4 for quantification of visceral adipose tissue (30 cm^2^) and subcutaneous adipose tissue (450 cm^2^). **b** CT of the mid-thigh for quantification of subcutaneous adipose tissue (208 cm^2^) and muscle (110 cm^2^). **c** 1H-MR spectrum of the right hepatic lobe for intrahepatic lipid quantification showing lipid (1.3 ppm) and unsuppressed water (4.7 ppm) resonances. Lipid to water ratio was 0.01. For purposes of visual comparison, the amplitude of unsuppressed water in Figs. 1c and 2c were scaled identically. **d** 1H-MR spectrum of soleus muscle for intramyocellular lipid quantification showing intramyocellular lipid methylene protons (-CH2) at 1.3 ppm (IMCL), extramyocellular lipid methylene protons (-CH2) at 1.5 ppm (EMCL), residual water peak at 4.7 ppm, and creatine (-CH2) resonance at 3.0 ppm. IMCL/unsuppressed water ratio was 0.02. For purposes of visual comparison, the amplitude of total creatine peaks in Figs. 1d and 2d were scaled identically

Men had higher mean VAT and VAT/SAT compared to women while women had higher thigh SAT and men higher thigh muscle CSA, independent of age and BMI. There was a trend toward higher mean abdominal SAT in women, which became significant after controlling for age and BMI. (Figs. 1 and 2, and Table 1).

### Sex differences in body composition as assessed by proton MR spectroscopy (1H-MRS)

There was a trend of higher mean IHL in men, which became significant after controlling for age and BMI. Men also had higher mean soleus IMCL compared to women, independent of age and BMI (Figs. 1 and 2, Table 1).

### Sex differences in cardiometabolic risk markers

Cardiometabolic risk markers of men and women are shown in Table 2. At similar mean age and BMI, men had a more detrimental cardiometabolic risk profile with impaired measures of glucose homeostasis (higher mean fasting insulin and HOMA-IR), serum lipids (lower mean HDL cholesterol, higher triglycerides, higher ApoB, and ApoB/LDL), and higher fibrinogen than women. Despite similar mean age and BMI, there was a higher prevalence of the metabolic syndrome in men compared to women (Table 2).

### Sex-specific associations of body composition and measures of cardiometabolic risk

Separate analyses of men and women revealed sex-specific differences in the associations between measures of body composition and cardiometabolic risk, which were independent of age and BMI.

VAT was associated with measures of impaired glucose homeostasis (fasting glucose, 2-h glucose, fasting insulin, HOMA-IR), serum lipids (total, HDL, and LDL cholesterol, triglycerides, ApoB), and inflammatory markers (hsCRP, fibrinogen) in men and women; however, many associations were stronger in women or not significant in men (Table 3).

**Table 3:** Relationship between visceral adipose tissue and cardiometabolic risk markers

	Visceral adipose tissue			
	Men		Women	
	*r*	*p*	*r*	*p*
Fasting glucose	0.30	0.003^#^	0.23	0.02^#^
2-h glucose	0.18	0.08*	0.35	0.0001*^#^
Fasting insulin	0.21	0.11*	0.46	0.002*^#^
HOMA-IR	0.21	0.1*	0.35	0.002*^#^
Total cholesterol	0.16	0.1^#^	0.33	0.0004^#^
HDL cholesterol	− 0.27	0.008*^#^	− 0.27	0.004*
LDL cholesterol	0.15	0.2*^#^	0.34	0.0002*^#^
Triglyceride level	0.28	0.006*^#^	0.39	< 0.0001*^#^
Apolipoprotein B (ApoB)	0.20	0.1^#^	0.37	0.001*^#^
ApoB/LDL	0.15	0.3	0.2	0.09
hsCRP	0.34	0.01*^#^	0.62	< 0.0001*^#^
Fibrinogen	0.42	0.001*^#^	0.34	0.003*

*Abbreviations*: *HOMA-IR* homeostatic model assessment of insulin resistance, *HDL* high-density lipoprotein, *LDL* low-density lipoprotein, *hsCRP* high-sensitivity C-reactive protein

*Significant after controlling for age

^#^Significant after controlling for BMI

IHL was associated with cardiometabolic risk markers in men and women; however, there were sex-specific differences. The associations between IHL and fasting glucose, apolipoprotein B, and fibrinogen were significant in women but not in men, while the associations between IHL and HDL cholesterol and hsCRP were significant in men but not in women (Table 4).

**Table 4:** Relationship between ectopic fat depots and cardiometabolic risk markers

	Intrahepatic lipids				Soleus intramyocellular lipids			
	Men		Women		Men		Women	
	*r*	*p*	*r*	*p*	*r*	*p*	*r*	*p*
Fasting glucose	0.15	0.3	0.32	0.01*^#^	0.12	0.3	0.007	1.0
2-h glucose	0.30	0.02*	0.40	0.001*^#^	0.08	0.5	− 0.09	0.5
Fasting insulin	0.62	< 0.0001*^#^	0.61	< 0.0001*^#^	0.15	0.3	− 0.01	1.0
HOMA-IR	0.59	0.0003*^#^	0.60	< 0.0001*^#^	0.16	0.2	− 0.02	0.9
Total cholesterol	0.09	0.5	0.20	0.1	0.10	0.4	− 0.09	0.5
HDL cholesterol	− 0.44	0.0003*^#^	− 0.19	0.1*	− 0.02	0.9	0.11	0.4
LDL cholesterol	0.10	0.5	0.14	0.3	0.06	0.6	− 0.15	0.3
Triglyceride level	0.39	0.002*^#^	0.57	< 0.0001*^#^	0.13	0.3	− 0.03	0.8
Apolipoprotein B (ApoB)	0.18	0.3	0.34	0.03*^#^	0.12	0.4	− 0.21	0.2
ApoB/LDL	0.47	0.006^#^	0.42	0.007*^#^	0.12	0.4	− 0.03	0.8
hsCRP	0.47	0.01*^#^	0.37	0.09	0.31	0.05*	0.41	0.06
Fibrinogen	0.21	0.2	0.50	0.001*^#^	0.44	0.002*	0.15	0.4

*Abbreviations*: *HOMA-IR* homeostatic model assessment of insulin resistance, *HDL* high-density lipoprotein, *LDL* low-density lipoprotein, *hsCRP* high-sensitivity C-reactive protein

*Significant after controlling for age

^#^Significant after controlling for BMI

IMCL were positively associated with inflammatory markers (fibrinogen, hsCRP) in men but not in women (Table 4).

Lower extremity fat mass/total fat mass was associated with favorable measures of glucose homeostasis (2-h glucose, fasting insulin, HOMA-IR) and serum lipids (triglycerides, ApoB, ApoB/LDL) in men and women, independent of age and BMI; however, many associations were stronger in women or not significant in men (Table 5).

**Table 5:** Relationship between body composition and cardiometabolic risk markers

	Lower extremity fat/total fat				Appendicular lean mass/weight			
	Men		Women		Men		Women	
	r	p	r	p	r	p	r	p
Fasting glucose	0.0002	1.0	− 0.17	0.08*^#^	− 0.20	0.06*^#^	− 0.20	0.03
2-h glucose	− 0.16	0.2*	− 0.36	0.0002*^#^	− 0.08	0.5	− 0.14	0.1
Fasting insulin	− 0.26	0.05*^#^	− 0.40	0.0005*^#^	− 0.48	< 0.0001*^#^	− 0.16	0.2
HOMA-IR	− 0.24	0.06*^#^	− 0.40	0.0004*^#^	− 0.49	< 0.0001*^#^	− 0.17	0.2
Total cholesterol	− 0.09	0.4	− 0.13	0.2	0.03	0.8	− 0.21	0.02^#^
HDL cholesterol	0.22	0.05*	0.10	0.3	0.13	0.2	0.08	0.4*
LDL cholesterol	− 0.10	0.4	− 0.09	0.4	0.07	0.5	− 0.19	0.04^#^
Triglyceride level	− 0.20	0.08	− 0.25	0.01*^#^	− 0.14	0.2	− 0.24	0.009
Apolipoprotein B (ApoB)	− 0.10	0.5	− 0.25	0.03*^#^	0.09	0.5	− 0.21	0.06^#^
ApoB/LDL	− 0.20	0.1	− 0.27	0.02*	− 0.13	0.3	− 0.14	0.2
hsCRP	− 0.08	0.6	− 0.17	0.3	− 0.40	0.003*	− 0.35	0.02
Fibrinogen	− 0.29	0.02*	− 0.09	0.4	− 0.41	0.001*	− 0.37	0.0008*

*Abbreviations*: *HOMA-IR* homeostatic model assessment of insulin resistance, *HDL* high-density lipoprotein, *LDL* low-density lipoprotein, *hsCRP* high-sensitivity C-reactive protein

*Significant after controlling for age

^#^Significant after controlling for BMI

Appendicular lean mass/weight was associated with favorable measures of glucose homeostasis (fasting insulin, HOMA-IR) in men, improved lipid profile in women (total cholesterol, LDL cholesterol, triglycerides, ApoB), and lower inflammatory markers (hsCRP, fibrinogen) in both sexes (Table 5).

## Discussion

Our study demonstrated differences in body composition between men and women with overweight/obesity. While women had a higher percent total fat mass and men more lean and muscle mass, detailed assessment of ectopic fat compartments revealed higher VAT, IHL, and IMCL in men while women had more lower extremity fat. At similar age and BMI, this male anthropometric phenotype was associated with a more detrimental cardiometabolic risk profile compared to the female phenotype. However, VAT was more strongly associated with measures of adverse cardiometabolic risk in women compared to men, while IMCL were more detrimental in men. Interestingly, relatively higher lower extremity fat mass was associated with a more favorable cardiometabolic risk profile and this was stronger in women than in men. Relatively higher appendicular lean mass was protective against cardiometabolic risk, and this was seen in both sexes.

There has been great interest in physiologic differences between men and women and the risk of cardiometabolic disease. The incidence and health outcomes in cardiometabolic disease differ between the sexes with men having a higher prevalence of cardiometabolic disease. However, although mortality is higher in men than women across the weight spectrum, the sex-specific increase in mortality is greater in women than men as BMI increases [4, 5]. This may be at least in part related to sex-specific differences in body composition. While women have relatively more fat mass and men more lean mass, less is known about sex differences in ectopic fat depots and their impact on cardiometabolic risk. Advances in imaging technology allow the comprehensive assessment of different fat compartments, including ectopic lipids, lean, and muscle mass [9–15]. A unique aspect of our study is the assessment of body composition by a combination of anatomic and functional imaging techniques. We used DXA to determine total body and appendicular fat and lean mass. However, DXA is not able to accurately quantify VAT and SAT. We therefore used CT to assess VAT, SAT, and thigh muscle. 1H-MRS has been shown to be an accurate technique to measure IHL and IMCL non-invasively [12–15], and we were able to quantify IHL and IMCL in our subjects with overweight/obesity.

Our study showed higher VAT, a strong risk factor for impaired glucose homeostasis, dyslipidemia, and the metabolic syndrome [7, 27], in men compared to women despite similar age and BMI. This is consistent with the propensity of men to accumulate fat in the abdomen (apple-shaped body type) while women had more lower extremity fat mass (pear-shaped body type). However, when we analyzed women and men separately, VAT was more strongly associated with markers of cardiometabolic risk in women compared to men. This is consistent with a study from the Framingham Heart cohort, in which VAT was more strongly associated with cardiometabolic risk factors in women compared to men [16]. This suggests that although women have less VAT than men overall, VAT accumulation in women confers greater cardiometabolic risk compared to men.

An important complication of obesity is elevated IHL content, which can lead to nonalcoholic fatty liver disease (NAFLD) and nonalcoholic steatohepatitis (NASH), which may progress to liver fibrosis and cirrhosis [6]. In our study, men had higher age-and BMI-adjusted IHL, assessed by a simple breath hold 1H-MRS sequence, compared to women of similar age and BMI. Men also had higher IMCL, which may play an etiologic role in the pathogenesis of insulin resistance. A recent study in lean men who underwent overfeeding for 8 weeks suggested that the size and location of lipid droplets, rather than the total IMCL content, are determinants of the increase in insulin resistance in this setting [28]. However, high IMCL content as determined by 1H-MRS has been shown in states of insulin resistance, type 2 diabetes mellitus (T2DM), and dyslipidemia [29]. Our finding of higher IMCL in men is consistent with a study by Machann et al. who assessed sex differences in body composition in 150 healthy volunteers across a wide age range who were at risk for developing T2DM [17].

Interestingly, IMCL were associated with higher inflammatory markers in men but not in women. We also found sex-specific differences in associations between IHL and cardiometabolic risk markers. While IHL was positively associated with fasting glucose, apolipoprotein B, and fibrinogen in women but not in men, IHL was associated with lower HDL cholesterol and higher hsCRP in men but not in women. This suggests that accumulation of IHL has different effects on glucose and lipid metabolism between the sexes.

Preferential accumulation of fat in the lower body (femorogluteal fat) has been shown to be relatively protective against cardiometabolic risk [7, 8, 30]. Consistent with this, we found more lower extremity fat in women than in men. Our observed more favorable cardiometabolic risk profile in women compared to men, despite similar age and BMI, might be in part due to greater lower extremity fat mass in women. Although lower extremity fat mass has been shown to be protective against cardiometabolic disease, sex differences between lower extremity fat and cardiometabolic risk are unknown. In our study, relative higher lower extremity fat mass was associated with more favorable measures of cardiometabolic risk and these associations were stronger in women than in men.

In our study, men had a worse cardiometabolic risk profile with impaired measures of glucose homeostasis, dyslipidemia and increased inflammatory markers and higher prevalence of the metabolic syndrome than women despite similar age and BMI. This is consistent with a study in premenopausal women and men of similar age, in which women were found to have more total body fat but lower VAT than men, which was associated with a more favorable cardiometabolic risk profile [31].

Skeletal muscle plays an important role in the regulation of glucose homeostasis [32], and low muscle mass contributes to increased risk of T2DM [33, 34]. Although men are known to have more muscle mass than women [1], less is known about sex differences in muscle mass and cardiometabolic risk. We found sex differences in appendicular lean mass normalized over weight which was associated with improved measures of glucose homeostasis in men, a more favorable lipid profile in women, and lower inflammatory markers in both sexes. These findings suggest that increasing muscle mass may be able to offset some of the detrimental effects of ectopic fat in men and women with obesity independent of changes in fat mass.

Potential mechanism for sex differences in body composition and its relationship with cardiometabolic risk include modulation by sex steroids. For example, low estrogen levels, as in menopause, are associated with preferential accumulation of VAT and increased cardiometabolic risk [35, 36], and low testosterone in men can lead to visceral adiposity [37]. Moreover, results of recent genome wide association studies (GWAS) have identified sex-specific genetic determinants of fat accumulation [38].

A limitation of our study is the cross-sectional study design. Longitudinal data are necessary to assess whether sex-specific differences in ectopic fat depots will translate into higher incidence of cardiometabolic disease. Furthermore, our observed sex differences and the differences in cardiometabolic risk do not imply causality and may be multifactorial, including lifestyle (diet, exercise) and genetic variations.

## Conclusion

Body composition differs between men and women and the male pattern of fat distribution is associated with higher cardiometabolic risk markers compared to women of similar age and BMI; however, VAT in women, and IMCL in men, is more detrimental to cardiometabolic health, while lower extremity fat is relatively more protective in women than in men. IHL were detrimental to both sexes with sex-specific differences in associations between IHL and cardiometabolic risk markers. This suggests that detailed anatomic and functional imaging, rather than BMI, provides a more complete understanding of metabolic risk associated with sex differences in fat distribution.
